# Myeloma‐induced leucocytoclastic vasculitis

**DOI:** 10.1002/jha2.32

**Published:** 2020-07-07

**Authors:** Kanchana De Abrew, R. Ayto, J. Milnthorpe

**Affiliations:** ^1^ Portsmouth Hospital NHS Trust Hampshire UK; ^2^ Salisbury NHS Foundation Trust Salisbury UK

A 76‐year‐old female was diagnosed with IgA‐lambda myeloma (stage R‐ISS 1), 17% plasma cells on bone marrow biopsy, with cytogenetics identifying trisomy 12 plus del 11q. MRI confirmed disease infiltration at T11, T12, and L4. She commenced bortezomib, cyclophosphamide, and dexamethasone, achieving a borderline VGPR post cycle 2. A sacral skin ulcer appeared during cycle 2, which progressed despite holding treatment (Fig 2). Biopsy confirmed leucocytoclastic vasculitis, which is thought to be myeloma induced (Fig 1a,b). Cryoglobulins, autoimmune screen, and hepatitis virology were negative. On partial healing, cyclophosphamide and dexamethasone were reintroduced, the addition of thalidomide being not tolerated, with new skin lesions developing post cycle 5 despite static light chain and continuing response. Repeat biopsy confirmed ongoing vasculitis. Treatment was stopped due to intolerance and lesions had healed prior to chemotherapy cessation.

**FIGURE 1:**
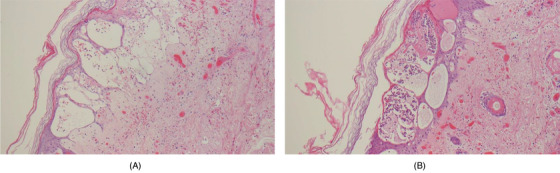
A and B, Skin biopsy. Necrosis within epidermis extending to subcutaneous fat. Damaged small and medium sized vessels within dermis and subcutaneous fat. Fibrin thrombi. Mixed inflammatory infiltrate with nuclear debris within dermis

**FIGURE 2:**
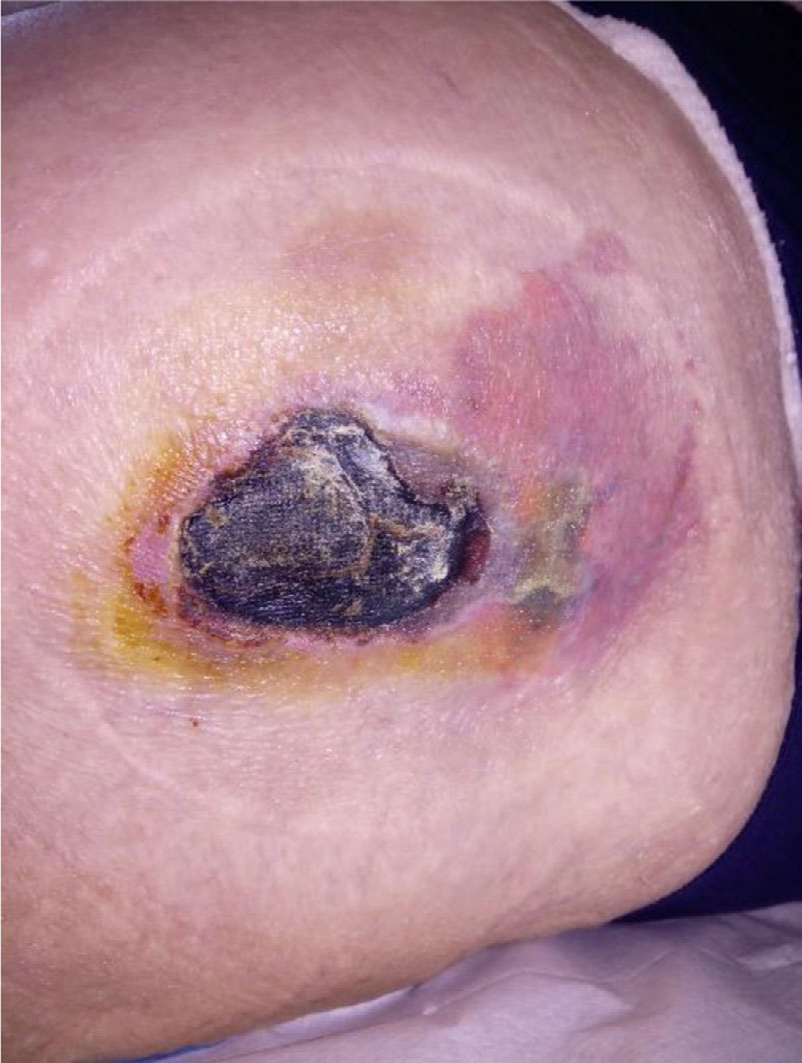
Necrotic nodule with surrounding purpura

Leucocytoclastic vasculitis affects small vessels including within the skin, with immune complex formation triggering complement deposition. Leucocytoclasia involves neutrophil breakdown following degranulation. This is rarely associated with myeloma, particularly IgA subtype, a prior series reporting eight cases within 2357 myeloma patients [1]. Triggers include infection, cryoglobulinaemia, drugs, and progressive disease. Disruptions of cell‐mediated immunity with cytokine overexpression (IL‐6, LFA‐1) are implicated [2]. Antimyeloma therapy is recommended together with dermatology input.

## Data Availability

Data are available on reasonable request from the authors.
